# Altered branched-chain **α**-keto acid metabolism is a feature of NAFLD in individuals with severe obesity

**DOI:** 10.1172/jci.insight.159204

**Published:** 2022-08-08

**Authors:** Thomas Grenier-Larouche, Lydia Coulter Kwee, Yann Deleye, Paola Leon-Mimila, Jacquelyn M. Walejko, Robert W. McGarrah, Simon Marceau, Sylvain Trahan, Christine Racine, André C. Carpentier, Aldons J. Lusis, Olga Ilkayeva, Marie-Claude Vohl, Adriana Huertas-Vazquez, André Tchernof, Svati H. Shah, Christopher B. Newgard, Phillip J. White

**Affiliations:** 1 Sarah W. Stedman Nutrition and Metabolism Center and Duke Molecular Physiology Institute, Duke University Medical Center, Divisions of Endocrinology and Cardiology, Department of Medicine, and Department of Pharmacology and Cancer Biology, Durham, North Carolina, USA.; 2 Institut Universitaire de Cardiologie et de Pneumologie de Québec, Université Laval, Quebec City, Quebec, Canada.; 3 Faculty of Medicine and Health Sciences, Centre de recherche du Centre hospitalier universitaire de Sherbrooke, Université de Sherbrooke, Sherbrooke, Quebec, Canada.; 4 Department of Medicine/Division of Cardiology, UCLA, California, USA.; 5 Facultad de Química, Universidad Nacional Autónoma de México/Instituto Nacional de Medicina Genómica, Unidad de Genómica de Poblaciones Aplicada a la Salud, Mexico City, Mexico.; 6 Centre de Nutrition, Santé et Société and Institute of Nutrition and Functional Foods, Université Laval, Quebec City, Quebec, Canada.

**Keywords:** Hepatology, Metabolism, Amino acid metabolism, Obesity

## Abstract

Hepatic de novo lipogenesis is influenced by the branched-chain α-keto acid dehydrogenase (BCKDH) kinase (BCKDK). Here, we aimed to determine whether circulating levels of the immediate substrates of BCKDH, the branched-chain α-keto acids (BCKAs), and hepatic *BCKDK* expression are associated with the presence and severity of nonalcoholic fatty liver disease (NAFLD). Eighty metabolites (3 BCKAs, 14 amino acids, 43 acylcarnitines, 20 ceramides) were quantified in plasma from 288 patients with bariatric surgery with severe obesity and scored liver biopsy samples. Metabolite principal component analysis factors, BCKAs, branched-chain amino acids (BCAAs), and the BCKA/BCAA ratio were tested for associations with steatosis grade and presence of nonalcoholic steatohepatitis (NASH). Of all analytes tested, only the Val-derived BCKA, α-keto-isovalerate, and the BCKA/BCAA ratio were associated with both steatosis grade and NASH. Gene expression analysis in liver samples from 2 independent bariatric surgery cohorts showed that hepatic *BCKDK* mRNA expression correlates with steatosis, ballooning, and levels of the lipogenic transcription factor *SREBP1*. Experiments in AML12 hepatocytes showed that SREBP1 inhibition lowered *BCKDK* mRNA expression. These findings demonstrate that higher plasma levels of BCKA and hepatic expression of *BCKDK* are features of human NAFLD/NASH and identify SREBP1 as a transcriptional regulator of *BCKDK*.

## Introduction

Nonalcoholic fatty liver disease (NAFLD) is characterized by neutral lipid accumulation in the liver. In approximately 1 of every 5 cases, this is accompanied by pathologic inflammation and hepatocellular damage (ballooning), termed nonalcoholic steatohepatitis (NASH) (1). This more pathogenic form of NAFLD progresses to fibrosis in approximately 35% of patients, significantly raising the risk for development of hepatocellular carcinoma, cirrhosis, and acute liver failure. Advanced NAFLD is also a significant risk factor for development of type 2 diabetes and cardiovascular diseases (2, 3).

There has been a sharp increase in the incidence of NAFLD in recent years due to the obesity pandemic; this has resulted in 25% of the US population having a diagnosis of NAFLD. The incidence of NALFD-related liver failure is now comparable to hepatitis C as a primary reason necessitating liver transplantation (4). An individual’s propensity for developing NAFLD is dictated by a combination of genetics, lifestyle, diet, and insulin sensitivity (5, 6). Hepatic triglyceride pools are influenced by supply of adipose-derived nonesterified fatty acids (NEFAs) to the liver, hepatic de novo lipogenesis (DNL), NEFA export in VLDLs, and hepatic rates of β oxidation and ketogenesis. Importantly, metabolic flux studies show that high compared with low hepatic fat content in otherwise well-matched study participants with obesity is associated with 3-fold higher rates of DNL but no difference in adipose efflux of NEFAs or production of VLDLs (7). Thus, hepatic DNL appears to be one important distinguishing feature of NAFLD status in the setting of obesity. Coupling of β oxidation to the TCA cycle rather than ketogenesis may also be an underlying feature of people with NAFLD (8), although this has yet to be studied in a population that is well matched for obesity but discordant for NAFLD.

Our prior work identified a new function for the branched-chain α-keto acid dehydrogenase (BCKDH) kinase (BCKDK) and protein phosphatase, Mg2^+^/Mn2^+^-dependent 1K (PPM1K) as posttranslational regulators of ATP citrate lyase (ACLY), a key enzyme involved in hepatic DNL (9, 10). Whereas phosphorylation of BCKDH by BCKDK is inhibitory and promotes accumulation of branched-chain α-keto acids (BCKAs) and their cognate branched-chain amino acids (BCAAs) in plasma (9), phosphorylation of ACLY is activating and results in increased DNL via production of cytosolic acetyl CoA, which leads to the formation of the immediate DNL precursor and inhibitor of fatty acid oxidation, malonyl CoA (11, 12). Hepatic BCKDK levels are elevated in genetic models of obesity and following ingestion of diets high in fructose in rats, whereas PPM1K levels are low in these settings and increased during fasting (9, 10). Feeding of diets high in fructose induces expression of the lipogenic transcription factor carbohydrate responsive–element binding protein-β (ChREBP-β), and ChREBP-β overexpression is sufficient to increase *BCKDK* and decrease *PPM1K* expression in rat liver (9). In addition, adenovirus-mediated overexpression of recombinant BCKDK increases phosphorylation of ACLY and raises DNL by 2.5-fold in lean healthy rats (9). Conversely, inhibition of BCKDK with a small molecule, BT2, or adenovirus-mediated overexpression of recombinant PPM1K in livers of obese Zucker fatty rats lowers hepatic triglyceride content by more than 40% (9). These effects occur within 7 days of BCKDK inhibition or PPM1K overexpression, in the absence of changes in food intake or body weight, alongside robust lowering of circulating BCKA (9). We also found an association between hepatic *BCKDK* and *ChREBPβ* expression in liver samples from humans with NASH (9), but the relationship of the metabolites directly regulated by this axis (i.e., BCKA) to NAFLD has not been examined.

Thus, we postulated that circulating levels of BCKAs correlate with NAFLD status in people with obesity. We tested and validated this hypothesis in a well-characterized cohort of 288 bariatric surgery patients with severe obesity (BMI, >35 kg/m^2^) from the Quebec Heart and Lung Institute (QHLI) Obesity Biobank, from whom liver biopsies were also taken at the time of bariatric surgery for histological grading of steatosis, inflammation, ballooning, and fibrosis. We also examined associations of hepatic *BCKDK* mRNA expression with hepatic steatosis, ballooning, and inflammation in 2 independent bariatric surgery cohorts. These studies highlighted a possibly novel relationship between *BCKDK* and the lipogenic transcription factor sterol regulatory–element binding protein-1 (SREBP1) that was subsequently explored in vitro.

## Results

### Characteristics of the metabolite study population.

To test our hypothesis that circulating levels of BCKAs are associated with NAFLD status in people with obesity we performed targeted metabolomic analysis in plasma samples from 288 bariatric surgery patients with severe obesity (BMI >35 kg/m2) from the QHLI Obesity Biobank; liver biopsies were also taken from these patients at the time of bariatric surgery for histological grading of steatosis, inflammation, ballooning, and fibrosis. Demographic and clinical information for the study population are provided in Table 1. The metabolite study population from the QHLI Obesity Biobank allowed for stratification by steatosis grade or NASH status among participants otherwise closely matched for age, sex, and BMI. With regards to clinical features associated with steatosis, we observed an increased prevalence of NASH and fibrosis in participants with more severe steatosis. We also genotyped participants for the presence of the patatin-like phospholipase domain-containing protein 3 (*PNPLA3*) Ile148Met variant, previously shown to be commonly associated with elevated liver fat in Mexican American individuals (13). At least 1 copy of the Ile148Met variant, or G allele, was present in 48% of the patients studied here. Consistent with prior reports in Mexican American individuals (13), the *PNPLA3* Ile147Met variant was more common in French Canadian individuals with higher steatosis grade; 27% of participants with a steatosis grade of 0 had at least 1 copy of this variant versus 78% of participants with a steatosis grade of 3. Steatosis grade was also associated with impaired fasting glucose, HbA1c, insulin, and fasting plasma glucose levels. No association was found between steatosis grade and the proportion of individuals taking medications for blood pressure or lipids. Steatosis grade was also not associated with total cholesterol, high-density lipoprotein, or low-density lipoprotein cholesterol concentrations, but it was strongly associated with plasma triglycerides and circulating liver enzyme levels (alanine transaminase [ALT], aspartate transaminase [AST], and γ-glutamyltransferase [GGT]).

With regards to the clinical features of NASH, the presence of NASH was associated with more severe steatosis, and individuals with NASH were more likely to have advanced fibrosis (grade 2–4) than those without NASH (38% vs. 13%). Associations between NASH and plasma lipids, liver enzymes, and medications mirrored those for steatosis grade. The *PNPLA3* Ile148Met variant, HbA1c, and fasting plasma glucose were also strongly associated with the presence of NASH.

### A BCKA-related signature of NAFLD status.

In order to test the association between BCKA and NAFLD status in the broader context of other potentially relevant metabolic pathways, we quantified 80 metabolites in the 288 plasma samples from the QHLI Obesity Biobank. The metabolites measured included the 3 BCKAs, 14 amino acids, 43 acylcarnitines, and 20 ceramides. Principal component analysis (PCA) with varimax rotation was employed for dimensionality reduction of these 80 metabolites, resulting in 18 PCA factors with eigenvalues of more than 1 and explaining 73% of total variance (Supplemental Table 1; supplemental material available online with this article; https://doi.org/10.1172/jci.insight.159204DS1). We then tested associations of the 3 individual BCKAs (α-keto-isovalerate [KIV], α-keto-isocaproate [KIC], and α-keto-β-methylvalerate [KMV]), the molar sum of BCAA (Val, Ile/Leu), the BCKA/BCAA ratio (molar sum of KIV, KIC, and KMV divided by the molar sum of Val, Ile/Leu), and the 18 PCA factors with steatosis grade, NASH, and the presence of advanced fibrosis using univariate and multivariate regression.

In univariate analyses, only KIV (the BCKA derived from Val) and the standardized BCKA/BCAA ratio were strongly associated with both steatosis grade and NASH (Table 2). Specifically, higher levels of KIV and larger BCKA/BCAA ratios were associated with higher steatosis grade (OR [95% CI], 1.2 [1.1–1.3], *P =* 1.1 × 10^–4^ and OR [95% CI], 1.5 [1.2–1.9], *P =* 2.9 × 10^–4^, respectively) and increased risk of NASH (OR [95% CI], 1.2 [1.1–1.3], *P =* 4.0 × 10^–4^ and OR [95% CI], 2.0 [1.5–2.7], *P =* 3.0 × 10^–6^, respectively). This association does not appear to be driven by BCAA, because none of the individual BCAAs or the PCA factor 5 (Supplemental Table 1), which is primarily composed of the 3 BCKAs and their cognate BCAA, displayed an association with steatosis grade or NASH.

Besides KIV and the BCKA/BCAA ratio, no other metabolite or metabolite factor was associated with both steatosis grade and NASH in univariate analysis. Moreover, of the 18 PCA factors listed in Supplemental Table 1, only factor 14 (alanine and proline) and factor 7 (glycine, serine, and histidine) were associated with steatosis grade, whereas factor 10 (C20:4, C22, C18:2-OH acylcarnitines) was the only PCA factor associated with NASH alone (Supplemental Table 1). No metabolites or factors measured in the present study were found to be associated with the presence of advanced fibrosis.

The associations of KIV with steatosis grade and NASH, and the BCKA/BCAA ratio with NASH, were maintained when a multivariate statistical model that considered BMI, sex, age, HbA1c, ALT, AST, GGT, *PNPLA3* Ile148Met genotype, and study batch was applied (Table 2). Similar results were obtained when insulin was used in place of Hba1c (data not shown). However, the associations of BCKA/BCAA ratio with steatosis grade and factor 10 with NASH were somewhat attenuated after adjustment for these clinical variables. To explore this further, we tested for 2-way interactions between sex and *PNPLA3* Ile148Met genotype and these metabolites. The interaction of the BCKA/BCAA ratio with sex was marginally significant (*P =* 0.05); sex-stratified analysis then revealed that BCKA/BCAA was associated with steatosis grade in female participants (OR [95% CI], 1.7 [1.2–2.3], *P =* 0.002), but the association was completely absent in the male cohort (OR [95% CI], 1.0 [0.7–1.5], *P =* 0.9) (Supplemental Figure 1). Similarly, we observed an interaction between factor 10 and the *PNPLA3* Ile148Met variant in the NASH analysis (*P =* 0.003). In stratified analyses, there was no association of factor 10 and NASH in carriers of the *PNPLA3* Ile148Met variant (G allele) (OR [95% CI], 0.8 [0.5–1.4], *P =* 0.5), while participants without a copy of the variant showed an association (OR [95% CI], 0.3 [0.1–0.6], *P =* 7 × 10^–4^) (Supplemental Figure 1).

### Association of hepatic BCKDK mRNA expression with features of NASH in people with severe obesity.

We next explored the relationship between *BCKDK* mRNA expression in hepatic tissue and the individual features of NASH, using liver biopsies from a smaller cohort of 60 bariatric surgery patients with severe obesity from the QHLI Obesity Biobank, as well as existing transcriptomics data from 107 bariatric surgery patients with severe obesity from the Mexican Obesity Surgery (MOBES) cohort (Supplemental Table 2). Despite the potential environmental differences and differences in ethnicity present among the French Canadian and Mexican cohorts, the relationships between hepatic *BCKDK* mRNA expression and features of NASH were remarkably similar (Table 3). Results from both cohorts were consistent with the strong associations observed for KIV BCKA/BCAA ratio with steatosis and NASH, showing significant positive associations between hepatic *BCKDK* gene expression and steatosis grade, ballooning, and NASH. There was no association between *BCKDK* expression and lobular inflammation in either cohort.

### The lipogenic transcription factor SREBP1 regulates BCKDK mRNA expression.

**Figure 1:**
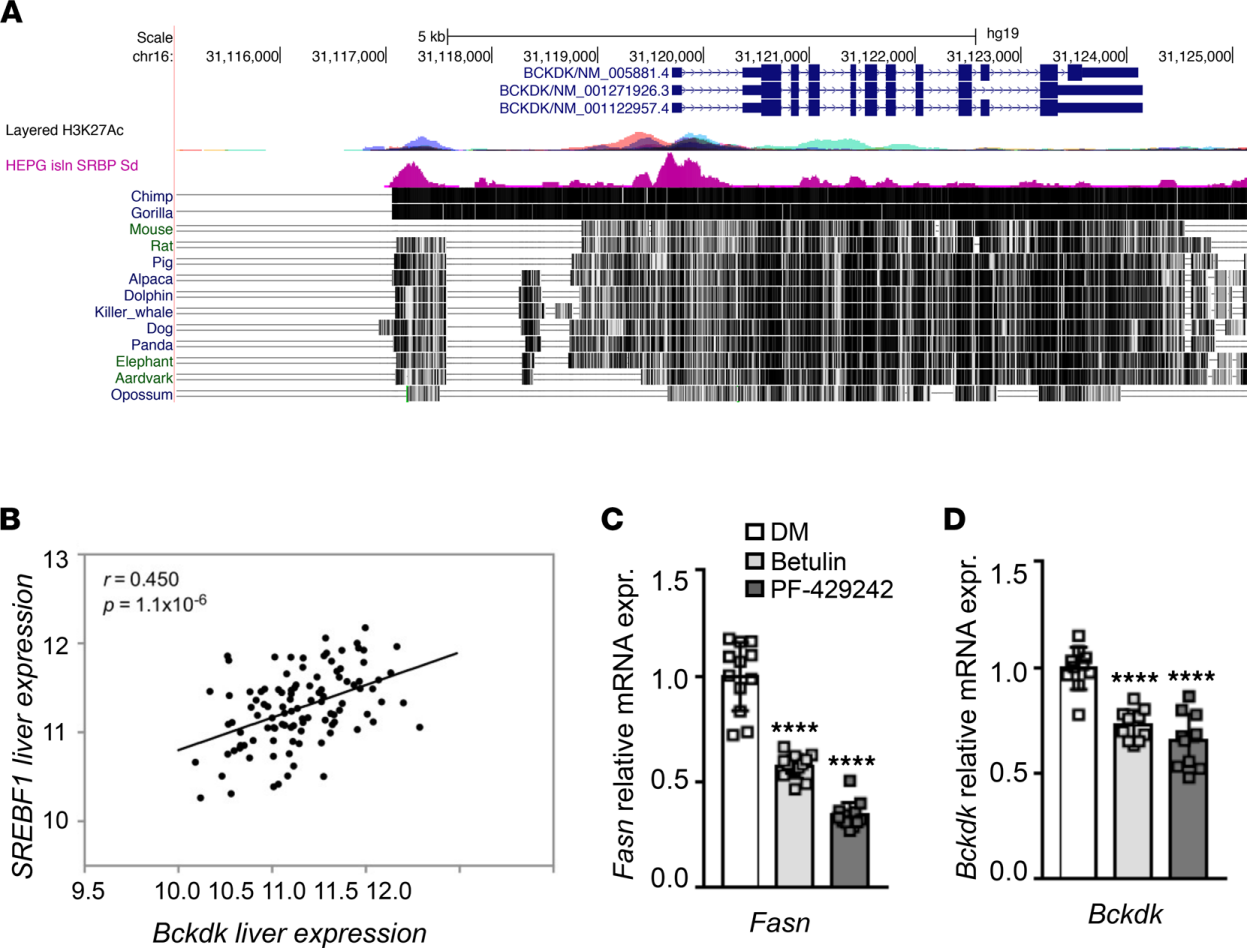
Regulation of *BCKDK* expression by SREBP1. (**A**) H3K27Ac peaks (multicolored) and SREBP1 ChIP-Seq data (pink) in the promoter and upstream enhancer region of the *BCKDK* gene. Conservation of these genomic regions relative to the human genome is indicated by vertical black hatch marks to the right of each mammal. (**B**) Correlation between *BCKDK* and *SREBP1* gene expression in liver samples from the Mexican Obesity Surgery cohort. (**C** and **D)** The effect of the SREBP1 inhibitors, Betulin and PF-429242, on *FASN* and *BCKDK* mRNA expression in AML12 cells. Data are expressed as the mean ± SEM of 3 independent experiments. A 1-way ANOVA with a Dunnett’s post hoc test was employed to determine statistical significance. ****P < 0.001.

Our prior work identified the fructose-sensing lipogenic transcription factor, ChREBP, as a mechanism linking BCKDK to the lipogenic process (9). In line with this prior observation, we observed a positive association between the *ChREBPα* and *BCKDK* transcript levels in the sequencing data from the MOBES cohort (R = 0.33, *P =* 6.4 × 10^–5^). In light of our observation of a strong association of circulating BCKA and hepatic *BCKDK* expression with NAFLD/NASH in people with severe obesity we aimed to determine whether another major lipogenic transcription factor, SREBP1 might also regulate hepatic *BCKDK* gene expression. Examination of SREBP1 ChIP-Seq data and histone acetylation patterns surrounding the *BCKDK* locus in HEPG2 cells in the ENCODE database (https://www.encodeproject.org/) revealed that SREBP1 peaks are present in both the *BCKDK* promoter and the enhancer region upstream of the *BCKDK* gene where ChREBP was previously found to bind (Figure 1A). Sequence alignment data show that, in contrast to the enhancer region identified as bound by ChREBP that is present in humans and rats but not mice, the promoter region bound by SREBP1 was conserved across all species (Figure 1A). In line with these ChIP-Seq data, examination of the relationship between *SREBP1* and *BCKDK* transcript levels in the RNA-Seq data from the MOBES cohort revealed a robust positive correlation between *SREBP1* and *BCKDK* mRNA expression in human liver (R = 0.45, *P =* 1.1 × 10^–6^; Figure 1B). We therefore directly tested the regulation of *BCKDK* mRNA expression by SREBP1 in AML12 hepatocytes in vitro using 2 well-validated chemical inhibitors of SREBP1, Betulin (14) and PF-429242 (15). Forty-eight hours after treatment with either Betulin or PF-429242, AML12 hepatocytes displayed significantly lower expression of the SREBP1 target gene, fatty acid synthase (*FASN*), as well as *BCKDK* (Figure 1, C and D). Together, these data place SREBP1 alongside ChREBP as a lipogenic transcriptional regulator of *BCKDK* gene expression. This finding is consistent with the well-established overlap between SREBP1 and ChREBP in the regulation of lipogenic gene expression; however, additional work is warranted to determine whether one of these lipogenic transcription factors exerts a stronger influence on hepatic BCKDK expression or if simultaneous binding of both factors exerts a synergistic effect on hepatic BCKDK induction in liver.

## Discussion

Our metabolomic analysis of plasma from a well-characterized population of people with severe obesity revealed that BCKA, KIV, and the BCKA/BCAA ratio were the only metabolite features strongly associated with both steatosis grade and NASH, even after adjustment for multiple covariables. Moreover, we demonstrated that the hepatic expression of *BCKDK* mRNA is strongly associated with steatosis grade, ballooning, and NASH in livers of 2 distinct bariatric surgery populations with severe obesity. These results are consistent with our finding in rats showing that pharmacologic or molecular manipulation of the hepatic BCKDK/PPM1K ratio to favor BCKDK simultaneously increases circulating BCKA levels, via inhibition of BCKDH, and hepatic DNL, via activation of ACLY (9). The current findings expand upon our studies in rodents to implicate this integrative metabolic regulatory node as a potential therapeutic target for human NAFLD. Our studies also introduce SREBP1 as a second lipogenic transcription factor, along with ChREBP-β, in regulation of *BCKDK* gene expression. Finally, our data provide evidence for a potential clinical utility for plasma levels of KIV or the BCKA/BCAA ratio to identify individuals with NAFLD/NASH that might be best suited to therapies that target the lipogenic machinery.

BCAAs have repeatedly emerged as strong biomarkers of cardiometabolic disease traits such as obesity, insulin resistance, and future diabetes development (10, 16–20). More recently, a number of studies have demonstrated an association between BCAA and presence of NAFLD (21–25). Herein, our work suggests that BCKAs are a more sensitive indicator of NAFLD and NASH status than BCAA in people with severe obesity. We propose that this is due to the fact that circulating BCAAs can be affected by a diverse set of metabolic adaptations in the obese milieu, including insulin resistance, dietary changes, altered gut microbiota, and downregulation of components of BCAA catabolism in metabolic tissues other than liver, such as adipose (10, 26–33). In contrast, the liver does not metabolize BCAA due to very low levels of the branched-chain amino-acid aminotransferase (BCAT) (34), whereas its high levels of BCKDH expression make it one of the most active sites of BCKA catabolism (35, 36). Thus, our data support the notion that measurement of plasma BCKAs or the ratio of BCKA/BCAA, which provides correction for systemic BCAA load, provides a sensitive index of the balance of BCKDK/PPM1K in the liver and NAFLD status.

Although the single BCKA KIV was strongly associated with both steatosis grade and NASH in both univariate and multivariate analyses, the association of the BCKA/BCAA ratio with steatosis grade was attenuated when the multivariate model was applied and, subsequently, determined to be significant only in female participants. This sex interaction may be driven by differences in the partitioning of BCAA. Indeed, levels of BCAA and related metabolites are known to be higher in male than female individuals, as shown in a cohort of adults who are overweight or obese (37) or in pediatric participants with a comparable BMI (38). Moreover, BCAA levels were recently reported to be associated with NAFLD status in women but not in men (24). Moreover, sex-dependent differences in the relationship of BCAA with fasting glucose and lipids have been described in early adolescence (39). One possible mechanism underlying these observations is that the female sex hormone estrogen promotes BCAA uptake by inducing the expression of the cell polarity protein LLGL7L2, which binds to and activates the large neutral amino acid transporter SLC7A5 at the cell surface (40). However, additional work is needed to better understand the differential regulation of BCAA utilization across the sexes and whether this contributes to any differences in risk for NAFLD and/or other cardiometabolic diseases.

Beyond KIV and the BCKA/BCAA ratio, we identified 2 additional amino acid–related factors associated with steatosis grade, as well as a long-chain acylcarnitine-related factor that was associated with the presence of NASH in carriers of the major *PNPLA3* allele. Factors 7 and 14 are made up of glycine-related amino acids (glycine, serine, and histidine) and nitrogen-handling metabolites (alanine and proline), respectively. Importantly, the negative association of glycine and serine with steatosis grade and BCAA levels observed here has also been reported in other cohorts (26, 36, 41–43). Factor 10, composed of C20:4, C22, C18:2 carnitines, was found to associate with the presence of NASH in univariate analysis. Given that the metabolite with strongest loading in this factor is arachidonyl (C20:4) carnitine, it is tempting to speculate that the association between this factor and NASH is driven by arachidonic acid–derived lipid mediators known to play a role in inflammation, such as the leukotrienes and prostaglandins (44).

In conclusion, this study provides proof of concept in humans that the BCKA plasma level or the BCKA/BCAA ratio associates with NAFLD status in obese individuals. This finding also provides support for the idea that excessive hepatic expression of the BCKDK, now understood to also play a role in regulation of the critical DNL enzyme ACLY (9), plays an important role in the development of NAFLD in human obesity. The major strengths of our study include the use of a well-characterized population matched for several confounders across grades of liver steatosis, the use of samples in which liver phenotypes were determined via gold standard histological grading, and validation of gene expression studies across obesity cohorts with different ethnic backgrounds. Future studies are warranted to verify if the associations described herein will be observed in other populations with different ethnic backgrounds and other forms of fatty liver disease.

## Methods

### Study participants.

The study population employed for plasma metabolomic analysis consisted of a cohort of patients of European ancestry with severe obesity (BMI >35 kg/m^2^) from the eastern provinces of Canada who underwent bariatric surgery at the Institut universitaire de cardiologie et de pneumologie de Québec (QHLI). Of the patients with available samples at the QHLI Obesity Biobank, 404 met the initial inclusion criteria for the study, which were as follows: HbA1c of less than 6% and fasting plasma glucose of less than 126 mg/dL, histologic NAFLD characterization, consent for genetic studies, and not on diabetes medications. NAFLD was present in 79% of these patients (steatosis grade 1 [5%–33%], 55%; grade 2 [34%–66%], 18%; grade 3 [>67%], 6%). For this study, 288 participants were selected across a range of steatosis grades (grade0–3) and were matched for age, sex, BMI, and glucose tolerance; additionally, participants had steatosis with NASH (defined as the presence of steatosis alongside both lobular inflammation and ballooning) or without NASH. Evaluation of liver histology was performed by a pathologist according to the methods of Brunt et al. (45). Biospecimens were obtained from the biobank of the Institut universitaire de cardiologie et de pneumologie de Québec in accordance with institutionally approved management modalities.

For liver gene expression analyses, a set of liver biopsy specimens from 60 individuals with severe obesity (BMI >35 kg/m^2^) was obtained from the QHLI Obesity Biobank. Similar to the samples used for metabolomic analysis, samples were from patients of European ancestry from the eastern provinces of Canada with severe obesity who underwent bariatric surgery at the QHLI and were discordant for NAFLD and NASH, as determined by a pathologist according to the methods of Brunt et al. (45). The results from this targeted gene expression analysis were validated using existing transcriptomic data from the MOBES cohort (46–48).

### Genotyping.

*PNPLA3* genotyping for the Ile148Met variant associated with hepatic steatosis (rs738409) was performed for samples from the QHLI Obesity Biobank on genomic DNA extracted from blood buffy coats using the GenElute Blood Genomic DNA kit (MilliporeSigma). rs738409 was genotyped using validated primers and TaqMan probes (Applied Biosystems). PNPLA3 genotypes were determined using 7500 Fast Real-Time PCR System (Applied Biosystems).

### Metabolite profiling.

BCKA, amino acid, acylcarnitine, and ceramide levels were measured in plasma by targeted metabolomics methods, as previously described (36, 49). Briefly, plasma concentrations of the α-keto acids of Leu (KIC, KMV, and KIV) were measured by LC-MS, and amino acid (*n =* 15), acylcarnitine (*n =* 45), and ceramide (*n =* 21) profiling was performed by tandem mass spectrometry. All MS analyses employed stable isotope dilution with internal standards from Isotec, Cambridge Isotopes Laboratories, and CDN Isotopes.

### Gene expression analysis.

For the human liver samples from the QHLI Obesity Biobank, PCR was used to measure gene expression. Specifically, total RNA was isolated using the total RNA purification kit (NORGEN BIOTEK). Total RNA for in vitro experiments was isolated using TRI-Reagent (MilliporeSigma, T9424). cDNA was generated using the high-capacity reverse transcription kit (Applied Biosystems), and real-time qPCR was performed on a QuantStudio 6 Flex Real-Time PCR system (Applied Biosystems) using specific primers (Supplemental Table 3) and the PowerUp SYBR Green Master Mix (Applied Biosystems), following the manufacturer’s instructions. Gene expression was normalized to RPLP0 using the ddCT method.

### RNA-Seq.

RNA isolation and sequencing for the MOBES cohort has been described previously (47, 48, 50). Briefly, sequencing was performed using an Illumina HiSeq2500 instrument. After data quality control, sequencing reads were mapped to the human reference genome using TopHat software v2.0.1 (51) and quantified using Cufflinks software (52).

### ChIP-Seq analysis in ENCODE.

The presence of SREBP1 ChIP peaks near the *BCKDK* transcription start site and previously identified ChREBP binding site upstream of the *BCKDK* gene was assessed using the HEPG2 SREBP1 Standard insulin ChIP-Seq Signal from the ENCODE/SYDH data set, available in the ENCODE project (GEO GSM935627) (53).

### AML12 experiments.

α Mouse liver cells (ATCC, CRL2254) were cultured in Ham’s F-12 (Gibco, ThermoFisher Scientific, 11320033) supplemented with 10% FBS (Gibco, ThermoFisher Scientific, 10437036), 1% glutamine (Gibco, ThermoFisher Scientific, 25030-081), 10 μg/mL insulin (Gibco, ThermoFisher Scientific, 12585014), 5 μg/mL transferrin (MilliporeSigma, T1147), 5 ng/mL selenium (MilliporeSigma, S9133), and 40 ng/mL dexamethasone (MilliporeSigma, D4902). They were maintained in a humidified incubator at 37°C under 5% CO_2_. Cells were plated in 24-well plates at a density of 80,000 cells/well. The next day, cells were treated with 10 μM Betulin (Cayman Chemical, 11041) (14), 10 μM PF-429242 (Cayman Chemical, 15140) (15), or DMSO in complete growth media for 48 hours before harvesting of mRNA in TRI-reagent for gene expression analysis.

### Statistics.

For the primary study of metabolomics in the samples from the QHLI Obesity Biobank, 80 metabolites met quality control standards and were used in PCA followed by varimax rotation; 18 independent PCA factors with an eigenvalue of more than 1 were retained for subsequent analysis and explained 73% of the total variance. Each PCA factor is described by its primary metabolites (|loading|>0.4) in Supplemental Table 1. In addition to PCA factors, we considered the 3 individual BCKAs (KIV, KIC, and KMV), BCAAs (Val, Ile/Leu), and the BCKA/BCAA ratio (molar sum of KIV, KIC, KMV/ molar sum of Val, Ile/Leu, standardized to give a quantity with mea*n =* 0 and SD *=* 1).

Proportional odds logistic regression was employed to test the association of the aforementioned variables with steatosis grade (grade 0, *n =* 57; grade 1, *n =* 118; grade 2, *n =* 58; grade 3, *n =* 55). Logistic regression was used to test the metabolite/PCA factor associations with NASH (yes [*n =* 74]/no [*n =* 202]) and advanced fibrosis (grade 0–1 [*n =* 230] vs. grade 2–4 [*n =* 57]). All associations were tested in both univariate models and multivariate models adjusted for HbA1c, liver enzyme levels (ALT, AST, GGT), BMI, sex, age, *PNPLA3* genotype, and technical batch. Associations were considered significant at *P <* 0.0024, given a Bonferroni adjustment for multiple tests. Metabolites/factors that were significantly associated with a phenotype in univariate, but not multivariate, models were tested for interactions with sex or genotype, followed by stratified analyses as appropriate. Statistical analyses were carried out in R v4.1.2.

For gene expression analyses in both the QHLI cohort and the MOBES cohort, a partial Pearson’s correlation analysis corrected for age, sex, and BMI was used to assess the association between *BCKDK* mRNA expression and the individual features of NASH. A nominal *P <* 0.05 was considered significant. Correlation between *BCKDK* and *SREBP1* expression within the MOBES transcriptomics data set was also assessed using a partial Pearson’s correlation.

The effects of the SREBP1 inhibitors Betulin and PF-429242 on *BCKDK* and *FASN* mRNA expression in AML12 cells were assessed using 1-way ANOVA with a Dunnett’s post hoc test. Data represent 3 independent experiments and are expressed as mean ± SD. A nominal *P* value of less than 0.05 was considered significant.

### Study approval.

All studies were conducted according to the principles outlined in the Declaration of Helsinki and approved by the institutional review boards at Université Laval, UCLA, Instituto Nacional de Medicina Genómica, and Duke University. All participants provided written, informed consent.

## Author contributions

PJW, TGL, and CBN conceptualized studies. PJW, CBN, SHS, AHV, LCK, YD, PLM, RWM, and TGL interpreted data. PJW, TGL, CBN, SHS, AT, AHV, AJL, RWM, and ACC planned studies. LCK, PLM, JMW, and YD performed statistical analyses. SM, ST, and CR acquired samples. OI conducted metabolomics analyses, MCV performed PNPLA3 genotyping. PLM analyzed sequencing data. YD conducted AML12 studies. PJW wrote the manuscript. CBN, SHS, LCK, TGL, AT, MCV, and PLM edited the manuscript. All authors read and approved the manuscript in its final form. Order of co–first authors was determined by seniority on the project.

## Supplementary Material

Supplemental data

## Figures and Tables

**Table 1:**
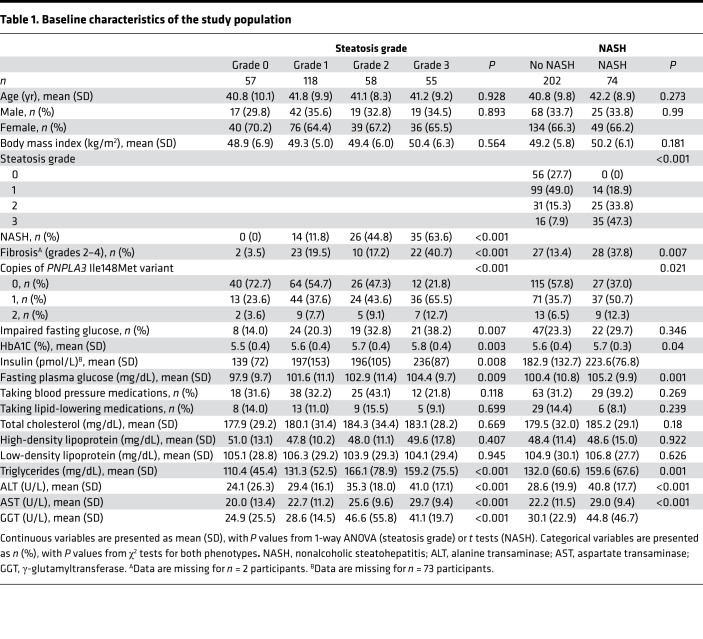
Baseline characteristics of the study population

**Table 2:**
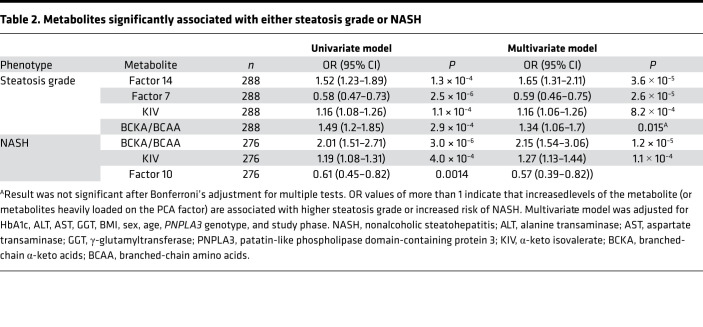
Metabolites significantly associated with either steatosis grade or NASH

**Table 3:**
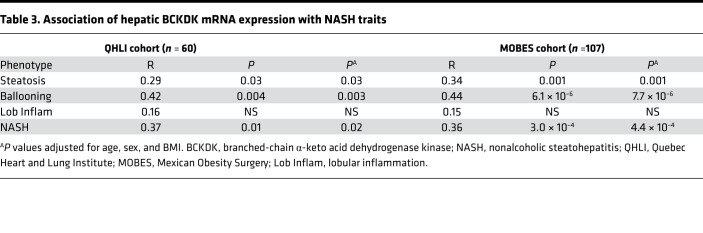
Association of hepatic BCKDK mRNA expression with NASH traits

## Version 1 07/07/2022

In-Press Preview

## Version 2 08/08/2022

Electronic publication
