# Supplementary material for: Altered branched-chain α-keto acid metabolism is a feature of NAFLD in individuals with severe obesity
Source: JCI Insight. 2022 Aug 8;7(15):e159204. doi: 10.1172/jci.insight.159204 (PMC9462486; doi:10.1172/jci.insight.159204)
Supplement: Supplemental data [file jciinsight-7-159204-s181.pdf]

SUPPLEMENTAL MATERIAL

Altered branched-chain α-keto acid metabolism is a feature of NAFLD in individuals with severe obesity

Thomas Grenier-Larouche[1,2,3*], Lydia Coulter Kwee[1*], Yann Deleye[1], Paola Leon-Mimila[4,5], Jacqueline M. Walejko[1], Robert W. McGarrah[1], Simon Marceau[2], Sylvain Trahan[2], Christine Racine[2], André C Carpentier[3], Aldons J. Lusis[4], Olga Ilkayeva[1], Marie-Claude Vohl[6], Adriana Huertas-Vazquez[4], André Tchernof[2], Svati H. Shah[1], Christopher B Newgard[1], Phillip J White[1#].

1. Sarah W. Stedman Nutrition and Metabolism Center and Duke Molecular Physiology Institute, Duke University Medical Center, Divisions of Endocrinology and Cardiology, Department of Medicine, and Department of Pharmacology and Cancer Biology, Durham NC USA.
2. Institut universitaire de cardiologie et de pneumologie de Québec - Université Laval (IUCPQ-UL) Québec City, QC, Canada
3. Faculty of Medicine and Health Sciences, Centre de recherche du Centre hospitalier universitaire de Sherbrooke, Université de Sherbrooke, Sherbrooke, QC, Canada.
4. Department of Medicine/Division of Cardiology, University of California, Los Angeles, California.
5. Facultad de Química, UNAM/Instituto Nacional de Medicina Genómica (INMEGEN), Unidad de Genómica de Poblaciones Aplicada a la Salud, Mexico City, Mexico.
6. Centre de Nutrition, santé et société and Institute of Nutrition and Functional Foods (INAF), Université Laval, Québec City, QC, Canada

Address correspondence to PJW: (phillip.white@duke.edu)

Table S1. PCA Factors

PCA Factor	Metabolite Components
**Factor 1 – Even chain acylcarnitines**	C14:1, C12:1, C2, C16:1,C14:2,C4-OH, C16:2,C14:1-OH, C12, C18:1, C6-DC/C8-OH, C16:1-OH/C14:1-DC, C14, C12-OH/C10-DC, C18:1-OH/C16:1-DC, C16, C10:1, C10, C14-OH/C12-DC, C18:2, C8, C8:1-DC, C18:1-DC
**Factor 2 – Glucosylceramides**	Glucosylceramides d18:1/C24, d18:1/C16, d18:1/C24:1, d18:1/C22, d18:1/C23, d18:1/C18, d18:1/C20; ceramide C16
**Factor 3 – Amino acid related**	Phenylalanine, leucine/isoleucine, methionine, arginine, tyrosine, valine, ornithine, C3 acylcarinitine, proline, citrulline, KMV, histidine
**Factor 4 – Ceramides**	Ceramides C26:1, C20, C26, C25, C18; glucosylceramide d18:1/C26
**Factor 5 – Branched-chain keto and amino acids**	KIC, KMV, KIV, leucine/isoleucine, valine
**Factor 6 – Medium chain OH/DC acylcarnitines**	C8:1-OH/C6:1-DC, C10-OH/C8-DC, C8:1-DC
**Factor 7 – Glycine related amino acids**	Glycine, serine, histidine
**Factor 8 – C18/C16 acylcarnitines**	C18, C16, C18:2, C18:1
**Factor 9 – Long chain ceramides**	Ceramides C24:1, C16, C20:1, C18
**Factor 10 – Long chain acylcarnitines**	C20:4, C22, C18:2-OH
**Factor 11 – Medium chain unsaturated acylcarnitines**	C8:1, C10:3, C10:2
**Factor 12 – Long chain ceramides**	Ceramides C23, C22, C24
**Factor 13 – Hydroxyisovaleryl/malonyl carnitine**	C5-OH/C3-DC
**Factor 14 – Alanine, proline**	Alanine, proline
**Factor 15 – C20 acylcarnitine**	C20
**Factor 16 – Short chain acylcarnitines**	C4/Ci4, C3, C5
**Factor 17 – Long chain dicarboxyl acylcarnitines**	C20-OH/C18-DC, C18:1-DC
**Factor 18 – Medium chain acylcarnitines**	C8, C10, C10:1

PCA factors were created using 80 metabolites. Top 18 factors displayed above (each with eigenvalue >1) explain 73% of total variance.

Table S2. Baseline characteristics of the gene expression cohorts

Trait	QHLI (N = 60)	MOBES (N = 107)
Age (Y)	45.0 (40.0 - 51.0)	35.0 (28.0 - 44.5)
Female (%)	42 (70)	93 (86.9)
BMI (kg/m2)	49.7 (46.6 - 52.7)	43.2 (39.1 - 48.5)
Glucose (mg/dL)	117.0 (99.0 - 146.7)	91.0 (80.3 - 99.8)
TG (mg/dL)	129.3 (100.7 - 159.0)	124.0 (91.0 - 162.0)
TC (mg/dL)	181.3 (162.3 - 203.5)	172.0 (147.0 - 194.0)
HDL-C (mg/dL)	48.7 (42.4 - 58.8)	36.0 (31.0 - 43.0)
LDL-C (mg/dL)	106.0 (83.2 - 121.0)	105.0 (86.6 - 129.0)
ALT (UI/L)	29.5 (21.0 - 41.0)	27.0 (21.0 - 38.0)
AST (UI/L)	23.0 (18.0 - 31.0)	26.0 (21.0 - 32.0)
GGT (UI/L)	29.0 (22.5 - 47.0)	19.0 (14.0 - 26.0)

Data are shown as median (interquartile range) or n (%). Abbreviations: BMI, Body Mass Index; TG, Triglycerides; TC, Total Cholesterol; HDL-C, high-density lipoprotein cholesterol; LDL-C, low-density lipoprotein; ALT, alanine aminotransferase; AST, aspartate aminotransferase; GGT, gamma-glutamyl transferase.

Table S3. Primers used for qPCR

Target	Forward Primers	Reverse Primers
**h*BCKDK***	5’ -TGAGAAGTGGGTGGACTTTGC- 3’	5’ -ATGGCATTCTTGAGCAGCTC- 3’
**m*Bckdk***	5’ -ATCTGTACTCGTCTGTCGCC- 3’	5’ -TCCGTTGATGCGGACTCG- 3’
**m*Fasn***	5’ -AGTCAGCTATGAAGCAATTGTGGA- 3’	5’ -CACCCAGACGCCAGTGTTC- 3’
RPLP0	5’ -TCTGCATTCTCGCTTCCTGG- 3’	5’-CCAGGACTCGTTTGTACCCG- 3’
